# Supplementary material for: Deep learning detects cardiotoxicity in a high-content screen with induced pluripotent stem cell-derived cardiomyocytes
Source: eLife. 2021 Aug 2;10:e68714. doi: 10.7554/eLife.68714 (PMC8367386; doi:10.7554/eLife.68714)
Supplement: Supplementary file 1. [file elife-68714-supp1.docx]

**Supplementary File 1.** Criteria for Defining Cardiotoxicity Scores and Binning Compounds Used to Establish Deep Learning Models

| **Cardiotoxicity Score** | **Description** | **Drug and**  **Condition Range^a^** | **Effect on Contractility** | **Sarcomere Damage** | **Nuclear Count** |
| --- | --- | --- | --- | --- | --- |
| Class 3 | Highly toxic | Bortezomib: 1 μM, 3 μM, 10 μM  Doxorubicin: 1 μM, 3 μM, 10 μM  Givinostat: 3 μM, 10 μM  Bafilomycin: 1 μM, 3 μM, 10 μM  Paclitaxol: 10 μM | Increase or decrease in beat rate > 30%  Displacement reduced by > 50%  Contraction velocity reduced by > 50% | No visible Z-disk, or highly damaged sarcomere present | < 20% cell survival by nuclear count |
| Class 2 | Toxic | Bortezomib: 0.03 μM, 0.1 μM, 0.3 μM  Cisapride: 1 μM, 3 μM, 10 μM  Sorafenib: 10 μM  Givinostat: 1 μM  Bafilomycin: 0.01 μM, 0.03 μM, 0.1 μM, 0.3 μM  Paclitaxol: 3 μM  JQ1: 10 μM | No significant effect on beat rate  Displacement reduced by > 35%  Contraction velocity reduced by > 35% | Visibly damaged sarcomeres | < 60% cell survival by nuclear count |
| Class 1 | Mildly toxic | Bortezomib: 0.01 μM  Doxorubicin: 0.3 μM  Cisapride: 0.3 μM  Givinostat: 0.3 μM  Paclitaxol: 1 μM  JQ1: 1 μM, 3 μM | No significant effect on beat rate  No significant effect on displacement  No significant effect on contraction velocity | Subtle damage to sarcomeres, not easily quantifiable | No significant effect on nuclear count |
| Class 0^b^ | Non-toxic | DMSO (0.1%) | No significant effect | No significant effect | No significant effect |

DMSO, dimethyl sulfoxide.

^a^Each drug concentration was binned to these classes based on three criteria: functional effects on contractility, extent of sarcomere damage, and number of surviving cells (nuclear count).

^b^DMSO-treated (0.1%) condition.
