# Supplementary material for: Deep learning detects cardiotoxicity in a high-content screen with induced pluripotent stem cell-derived cardiomyocytes
Source: eLife. 2021 Aug 2;10:e68714. doi: 10.7554/eLife.68714 (PMC8367386; doi:10.7554/eLife.68714)
Supplement: Supplementary file 2. [file elife-68714-supp2.docx]

**Supplementary File 2.** Criteria Used to Construct Classes of Deep Learning Models

| **Cardiotoxicity Score** | **Description** | **Number of Images^d^** | **4-Class Model^a^** | **3-Class Model^b^** | **2-Class Model^c^** | |
| --- | --- | --- | --- | --- | --- | --- |
| Class 3 | Highly toxic | 432 | Class 3 | Class 2 | Class 1 | |
| Class 2 | Toxic | 504 | Class 2 |  |  |  |
| Class 1 | Mildly toxic | 252 | Class 1 | Class 1 |  |  |
| Class 0 | Non-toxic | 504 | Class 0 | Class 0 | Class 0 | |

^a^Distinguishes highly toxic, toxic, and mildly toxic compounds from the non-toxic DMSO-treated condition (0.1%).

^b^Bins highly toxic and toxic compounds into a single category separate from mildly toxic compounds.

^c^Bins highly toxic and toxic compounds separate from the non-toxic DMSO-treated condition.

^d^The total number of images used per class (80% of images were used to construct the neural network; the remaining 20% were used to validate the deep learning model).
