# Supplementary material for: Deep learning detects cardiotoxicity in a high-content screen with induced pluripotent stem cell-derived cardiomyocytes
Source: eLife. 2021 Aug 2;10:e68714. doi: 10.7554/eLife.68714 (PMC8367386; doi:10.7554/eLife.68714)
Supplement: Supplementary file 3. [file elife-68714-supp3.docx]

**Supplementary File 3.** List of Compounds with Potential Structural Cardiotoxicity and Associated Information

| **Compound /Drug** | **Status** | **Indication/Pharmacological Classification** | **Primary Target** | **Reported Adverse Cardiovascular Events** |
| --- | --- | --- | --- | --- |
| WZ8040 | Tool compound | Oncology/EGFR inhibitor | EGFR |  |
| Zileuton | Approved. Immediate-release tablets withdrawn from the US market | Asthm, anti-inflammatory/5-lipoxygenase inhibitor | ALOX5 |  |
| Mosapride | Investigational drug (Phase 3) | Gastrointestinal disorders/5HT_4_ agonist | HTR4 |  |
| Roscovitine | Investigational drug (Phase 2) | Oncology, multiple indications/CDK2, CDK7, and CDK9 inhibitor | CDK2, CDK7, CKD9 |  |
| Streptozotocin | Approved | Pancreatic and other oncology, chemotherapy/alkylating antineoplastic agent | DNA |  |
| Adrucil | Approved | Oncology, chemotherapy/nucleoside metabolic inhibitor, DNA synthesis inhibitor | DNA | Side effects include cardiac toxicity (Adrucil. RxList., 2020) |
| Betamethasone | Approved | Topical steroid, immunosuppressive, anti-inflammatory/glucocorticoid receptor agonist | NR3C1 | Oral glucocorticoid identified as risk factor for heart failure (Souverein et al., 2004) |
| Meropenem | Approved | Broad-spectrum carbapenem antibiotic | dacB  (E. coli) |  |
| Regorafenib | Approved | Sorafenib analog, metastatic colorectal cancer/multi-kinase inhibitor | KIT, KRAS, BRAF, KDR | Risk of regorafenib-induced cardiovascular events in patients with solid tumors (Chen and Wang, 2018) |
| Lamivudine | Approved | Antiviral drug/HIV-1, HBV/reverse transcriptase inhibitor | DNA |  |
| Chlorpromazine | Approved | Antipsychotic medication, schizophrenia/dopamine and potassium channel inhibitor | DRD1, DRD2, KCNH2 | Fast/Irregular heart rate (Chlorpromazine HCL. WebMD., 2020) |
| Imidapril | Approved | Antihypertensive drug, ACE inhibitor | ACE |  |
| Rasagiline | Approved | Antidepressant, Parkinson’s disease/monoamine oxidase inhibitor | MAOB |  |
| Anagrelide | Approved | Blood thinner, Platelet-reducing agent/PDE3 inhibitor | PDE3 | Side effects may include fast, irregular, pounding, or racing heartbeat or pulse (Anagrelide. Mayo Clinic., 2020) |
| CI-1040 | Investigational drug (Phase 2) | Oncology, lung, breast, pancreatic, colorectal/MEK1, 2 inhibitor | MAP2K1  (MEK1),  MAP2K2  (MEK2) |  |
| Dimesna | Investigational drug (Phase 3) | Uroprotective agent used to decrease urotoxicity. Used as a chemoprotector in cisplatin-based chemotherapy |  | (Parker et al., 2010) |
| Sotalol | Approved | Anti-arrhythmic drug,  beta-blocker | ADRB1, ADRB2, KCNH2 | Serious side effects may include QT prolongation, heart failure, or bronchospasm (Sotalol. FDA.gov., 2020) |
| Melatonin | Approved | Insomnia, melatonin is a melatonin receptor agonist, used as a dietary supplement | MTNR1A, MTNR1B |  |
| Solifenacin | Approved | Overactive bladder with urinary incontinence/muscarinic receptor antagonist | CHRM3 | Symptoms of overdose may include fast heartbeat (Solifenacin. Medline Plus., 2020) |
| BI-2536 | Investigational drug (Phase 2) | Oncology/PLK1 and BRD4 Inhibitor | PLK1, BRD4 |  |
| Daunorubicin | Approved | Anthracycline. DNA and RNA synthesis and inhibits DNA synthesis/Topoisomerase II inhibitor | DNA, TOP2A | Doxorubicin analog, known to cause cardiotoxicity (Menna et al., 2012; Sawyer et al., 2010) |
| Nitrendipine | Approved | Antihypertensive agent, vasodilator/calcium channel blocker | CACNA2D1, CACNA1C, CACNB2 |  |
| PHA-767491 | Tool compound |  | CDK inhibitor |  |
| Rosiglitazone | Approved | Type 2 diabetes/  PPAR-γ agonist | PPARG | Associated with increase risk of myocardial infarction (Nissen and Wolski, 2007) |
| AG-1478 | Tool compound | EGFR inhibitor | EGFR |  |

ACE, angiotensin converting enzyme; ADRB, adrenoceptor beta; ALOX5, arachidonate 5-lipoxygenase; BRAF, B-Raf proto-oncogene, serine/threonine kinase; BRD, bromodomain containing.;CACNA/B, calcium voltage-gated channel auxiliary subunit (A/B); CDK, cyclin dependent kinase; CHRM, cholinergic receptor muscarinic; dacB, D-alanyl-D-alanine carboxypeptidase; DRD, dopamine receptor D; EGFR, epidermal growth factor receptor; HTR, 5-hydroxytryptamine receptor; KCNH, potassium voltage-gated channel subfamily H; KDR, kinase insert domain receptor; KIT, KIT proto-oncogene, receptor tyrosine kinase; KRAS, kirsten rat sarcoma; MAOB, monoamine oxidase B; MAP2K1, mitogen-activated protein kinase kinase; MTNR, melatonin receptor; NR3C, nuclear receptor subfamily 3 group C; PDE, phosphodiesterase; PLK, polo like kinase; PPARG, peroxisome proliferator activated receptor gamma; TOP2A, DNA topoisomerase II alpha.
