# Supplementary material for: Deep learning detects cardiotoxicity in a high-content screen with induced pluripotent stem cell-derived cardiomyocytes
Source: eLife. 2021 Aug 2;10:e68714. doi: 10.7554/eLife.68714 (PMC8367386; doi:10.7554/eLife.68714)
Supplement: Supplementary file 4. [file elife-68714-supp4.docx]

**Supplementary File 4.** Primary and Secondary Antibodies Used for Immunostaining

| **Type** | **Antibody** | **Dilution** | **Species** | **Manufacturer** | **Catalog Number** |
| --- | --- | --- | --- | --- | --- |
| Primary | Anti-MYBPC3 | 1:200 | Mouse (monoclonal) | Santa Cruz | Sc-137237 |
| Primary | Anti-ACTN2 | 1:200 | Rabbit (monoclonal) | Thermo Fisher Scientific | 701914 |
| Secondary | Donkey anti-Rabbit IgG (H+L)  Alexa Fluor 594 | 1:500 | Donkey anti-Mouse IgG (H+L) | Thermo Fisher Scientific | A-21202 |
| Secondary | Donkey anti-Mouse IgG (H+L)  Alexa Fluor 488 | 1:500 | Donkey anti-Rabbit IgG (H+L) | Thermo Fisher Scientific | A-21207 |
