# Supplementary material for: Deep learning detects cardiotoxicity in a high-content screen with induced pluripotent stem cell-derived cardiomyocytes
Source: eLife. 2021 Aug 2;10:e68714. doi: 10.7554/eLife.68714 (PMC8367386; doi:10.7554/eLife.68714)
Supplement: Supplementary file 5. [file elife-68714-supp5.docx]

**Supplementary File 5.** TaqMan qPCR Probes

| **Gene** | **Probe ID** | **Exon Boundary** | **Amplicon Length (bp)** | **Marker** |
| --- | --- | --- | --- | --- |
| *MYH7* | Hs01110632_m1 | 39-40 | 73 | Cardiac/Sarcomeric |
| *MYH6* | Hs01101425_m1 | 20-21 | 67 | Cardiac/Sarcomeric |
| *TNNI3* | Hs00165957_m1 | 7-8 | 93 | Cardiac/Sarcomeric |
| *TNNI1* | Hs00913333_m1 | 8-9 | 77 | Cardiac/Sarcomeric |
| *MYBPC3* | Hs00165232_m1 | 12-13 | 56 | Cardiac/Sarcomeric |
| *TNNT2* | Hs00943911_m1 | 14-15 | 152 | Cardiac/Sarcomeric |
| *GAPDH* | Hs99999905_m1 | 2-3 | 122 | Housekeeping |
