# Supplementary material for: Deep learning detects cardiotoxicity in a high-content screen with induced pluripotent stem cell-derived cardiomyocytes
Source: eLife. 2021 Aug 2;10:e68714. doi: 10.7554/eLife.68714 (PMC8367386; doi:10.7554/eLife.68714)
Supplement: Transparent reporting form [file elife-68714-transrepform.docx]

***eLife’s* transparent reporting form**

We encourage authors to provide detailed information *within their submission* to facilitate the interpretation and replication of experiments. Authors can upload supporting documentation to indicate the use of appropriate reporting guidelines for health-related research (see [EQUATOR Network](http://www.equator-network.org/%20)), life science research (see the [BioSharing Information Resource](https://biosharing.org/)), or the [ARRIVE guidelines](http://www.plosbiology.org/article/info:doi/10.1371/journal.pbio.1000412) for reporting work involving animal research. Where applicable, authors should refer to any relevant reporting standards documents in this form.

If you have any questions, please consult our Journal Policies and/or contact us: [editorial@elifesciences.org](mailto:editorial@elifesciences.org).

**Sample-size estimation**

- You should state whether an appropriate sample size was computed when the study was being designed
- You should state the statistical method of sample size computation and any required assumptions
- If no explicit power analysis was used, you should describe how you decided what sample (replicate) size (number) to use

Please outline where this information can be found within the submission (e.g., sections or figure legends), or explain why this information doesn’t apply to your submission:

Sample size is reported within each figure legend and we have indicated what the error bars denote.

**Replicates**

- You should report how often each experiment was performed
- You should include a definition of biological versus technical replication
- The data obtained should be provided and sufficient information should be provided to indicate the number of independent biological and/or technical replicates
- If you encountered any outliers, you should describe how these were handled
- Criteria for exclusion/inclusion of data should be clearly stated
- High-throughput sequence data should be uploaded before submission, with a private link for reviewers provided (these are available from both GEO and ArrayExpress)

Please outline where this information can be found within the submission (e.g., sections or figure legends), or explain why this information doesn’t apply to your submission:

Given that we wanted to mimic the conditions used in high throughput screening (HTS), we only performed each experiment once (1 biological replicate). In each HTS we used 8-16 positive and negative controls (all reported in figure legends). We then supplemented our findings by running follow-up validation studies and orthogonal assays. For each study / assay we ran between 8-40 technical replicates to ensure our findings were reproducible and can be used for statistical analysis. The number of replicates used in each study is reported in the figure legend.

For media optimization conditions we report data from 5 independent studies (biological replicates) in Figure 1­–figure supplement 1C,E & E to ensure reproducibility. This information is also reported in the figure legend.

**Statistical reporting**

- Statistical analysis methods should be described and justified
- Raw data should be presented in figures whenever informative to do so (typically when N per group is less than 10)
- For each experiment, you should identify the statistical tests used, exact values of N, definitions of center, methods of multiple test correction, and dispersion and precision measures (e.g., mean, median, SD, SEM, confidence intervals; and, for the major substantive results, a measure of effect size (e.g., Pearson's r, Cohen's d)
- Report exact p-values wherever possible alongside the summary statistics and 95% confidence intervals. These should be reported for all key questions and not only when the p-value is less than 0.05.

Please outline where this information can be found within the submission (e.g., sections or figure legends), or explain why this information doesn’t apply to your submission:

When appropriate in the figure legend we report number of replicates and P values (t-test). However, for high-throughput screening p values are not the most reliable method for analysis, instead we rely on Z-factor to look at model performance and measure assay variability and reproducibility.

Throughout this manuscript our error bars denote SD and is reported in each figure legend.

(For large datasets, or papers with a very large number of statistical tests, you may upload a single table file with tests, Ns, etc., with reference to sections in the manuscript.)

**Group allocation**

- Indicate how samples were allocated into experimental groups (in the case of clinical studies, please specify allocation to treatment method); if randomization was used, please also state if restricted randomization was applied
- Indicate if masking was used during group allocation, data collection and/or data analysis

Please outline where this information can be found within the submission (e.g., sections or figure legends), or explain why this information doesn’t apply to your submission:

Randomization not applicable in this manuscript

**Additional data files (“source data”)**

- We encourage you to upload relevant additional data files, such as numerical data that are represented as a graph in a figure, or as a summary table
- Where provided, these should be in the most useful format, and they can be uploaded as “Source data” files linked to a main figure or table
- Include model definition files including the full list of parameters used
- Include code used for data analysis (e.g., R, MatLab)
- Avoid stating that data files are “available upon request”

Please indicate the figures or tables for which source data files have been provided:

We deposited our RNA-Seq data on the Gene Expression Omnibus (GEO) database: GEO Submission (GSE172181),

<https://www.ncbi.nlm.nih.gov/geo/query/acc.cgi?acc=GSE172181>.

The MatLab code for scanning gradient Fourior transform (SGFT) is available from the following paper. This information is also reported in the M&M of the manuscript. The code for this SGFT algorithm is available on GitHub: https://github.com/maxsalick/SGFT.

Salick, M.R., Napiwocki, B.N., Kruepke, R.A., Knight, G.T., Ashton, R.S., Crone, W.C., 2020. The scanning gradient Fourier transform (SGFT) method for assessing sarcomere organization and alignment. J. Appl. Phys. 127, 194701. https://doi.org/10.1063/1.5129347
